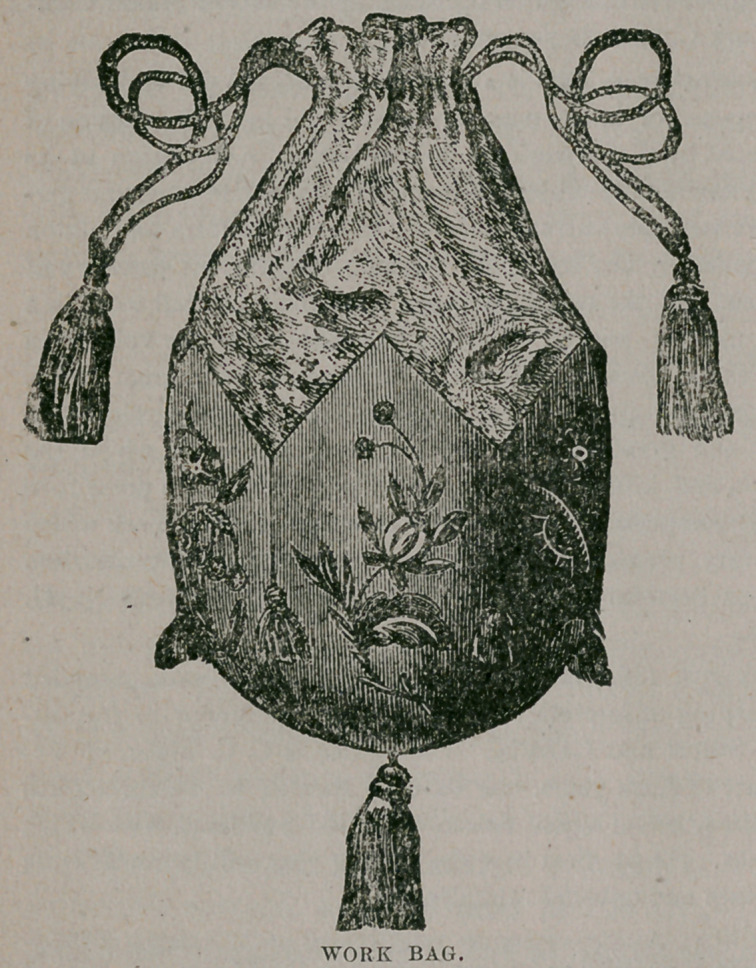# Household

**Published:** 1888-11

**Authors:** 


### HOUSEHOLD.

WORK BAG.—This little article, shown in our illustration, will be found very convenient for embroidery silks and needles. Cut the shape from stiff paper or an old hat frame, then cut the same shape of dark velvet and embroider in bright colors. When embroidered, press on wrong side only where the flowers are, else the nap of the velvet will flatten ; cover the stiff paper with the velvet, making sure that the edges are well turned over. Turn it wrong side out and overhand the points together at the bottom. Measure the size round the bag, then make an inside bag two and a half fingers in length allowing enough for a ruffle at the top. Finish with a cord and tassel in bright color to match the silk. The size can be suited to the purpose for which it is used, as a small bag will be large enough for silks, scissors, etc., while made quite a little larger the bag may contain the fancy work as well.

WORK BAG.

SPEAKING of apples, Prof. Faraday says : "There is scarcely an article of vegetable food more widely useful and more universally liked than the apple. Why every farmer has not an apple orchard, where the trees will grow at all, is one of the mysteries. Let every family, in autumn, lay in from two to ten or more barrels, and it will be to them the most economical investment in the whole range of culinary supplies. A raw, mellow apple is digested in an hour and a half, whilst boiled cabbage requires five hours. The most healthful dessert that can be placed on the table is baked apple. If taken freely at breakfast with coarse bread and butter, without meat or flesh of any kind, it has an admirable effect on the general system, often removing constipation, correcting acidities and cooling off febrile conditions more effectually than the most approved medicines. If families could be induced to substitute the apple—sound, ripe and luscious—for the pies, cakes, candies and other sweetmeats, with which children are too often stuffed, there would be a diminution of doctor's bills, sufficient in a single year to lay up a stock of this delicious fruit for a season's use."

## Figures and Tables

**Figure f1:**